# Supplementary figures and images for: Genome-Wide Characterization of Salt-Responsive miRNAs, circRNAs and Associated ceRNA Networks in Tomatoes
Source: Int J Mol Sci. 2021 Nov 12;22(22):12238. doi: 10.3390/ijms222212238 (PMC8625345; doi:10.3390/ijms222212238)

A

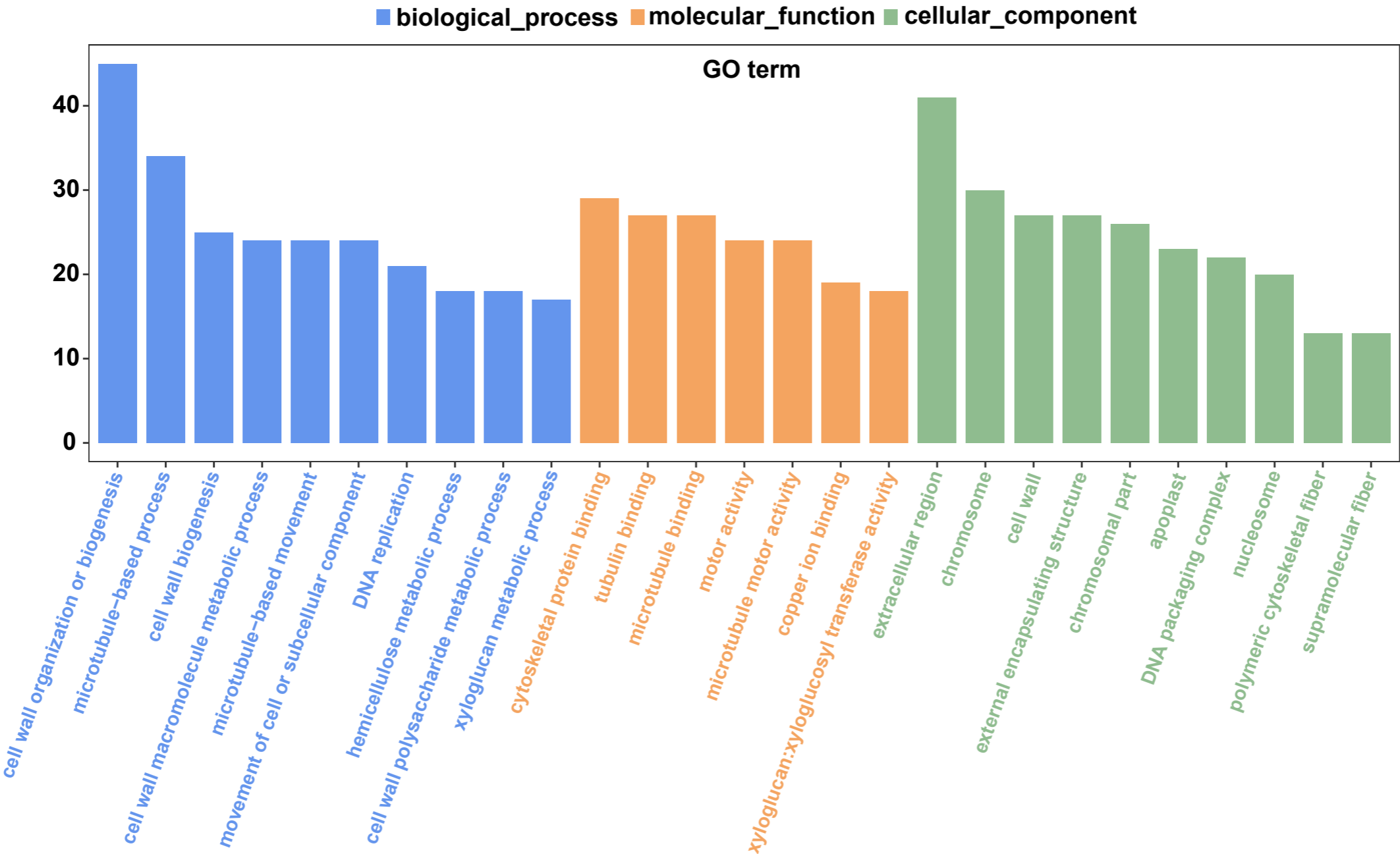

B

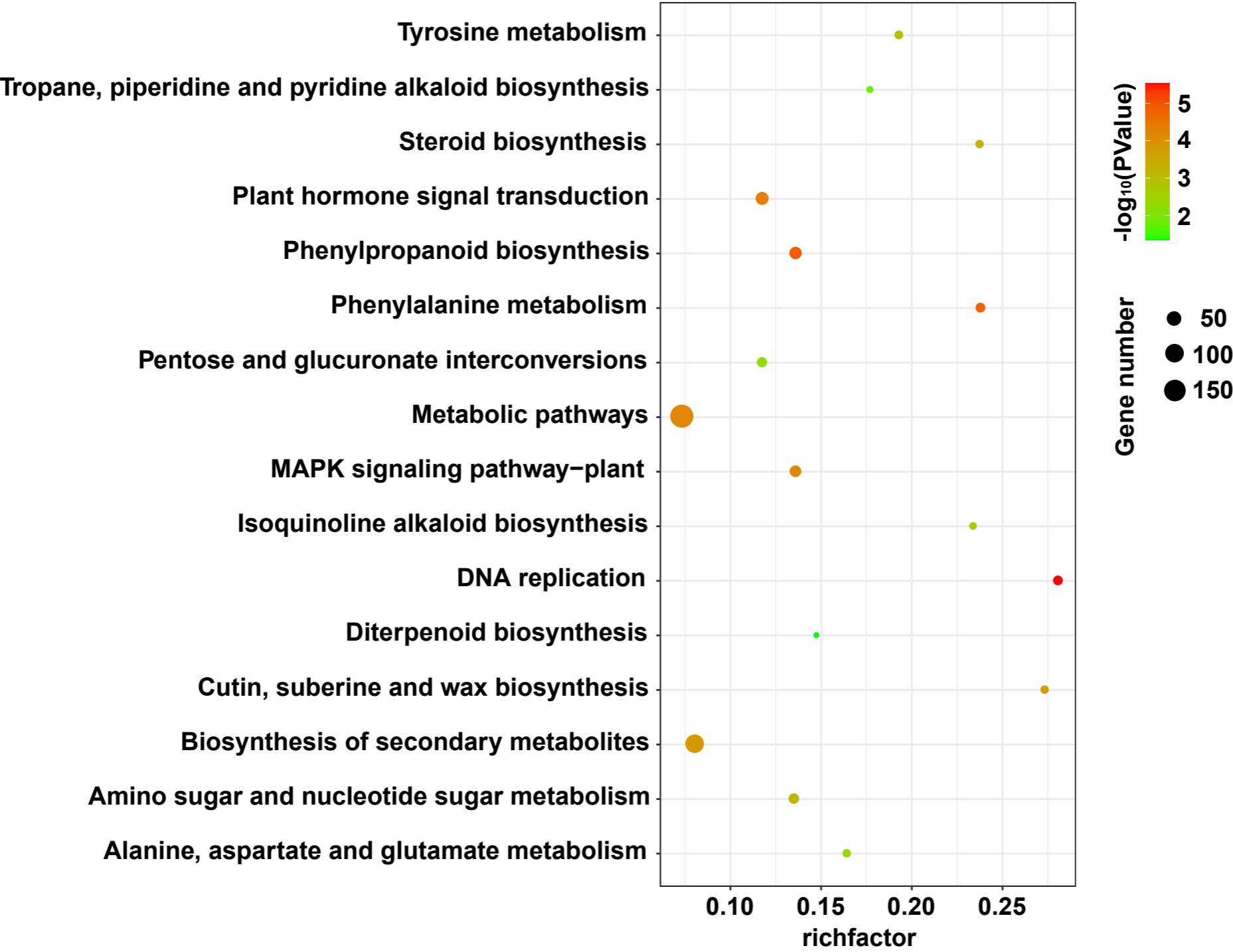

Supplement: Supplementary file 1 [file ijms-22-12238-s001.zip › Figure S1.pdf]

A

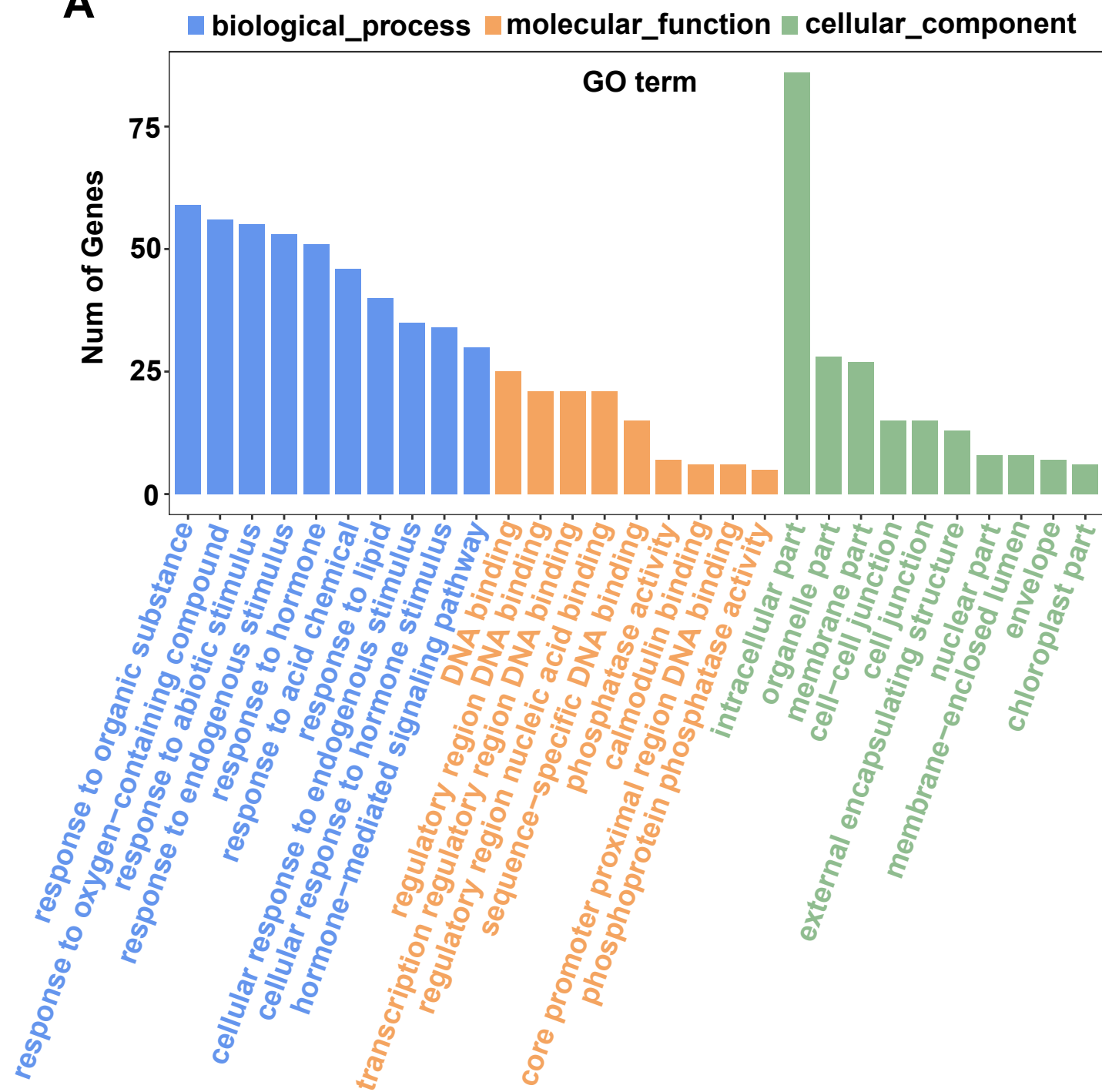

B

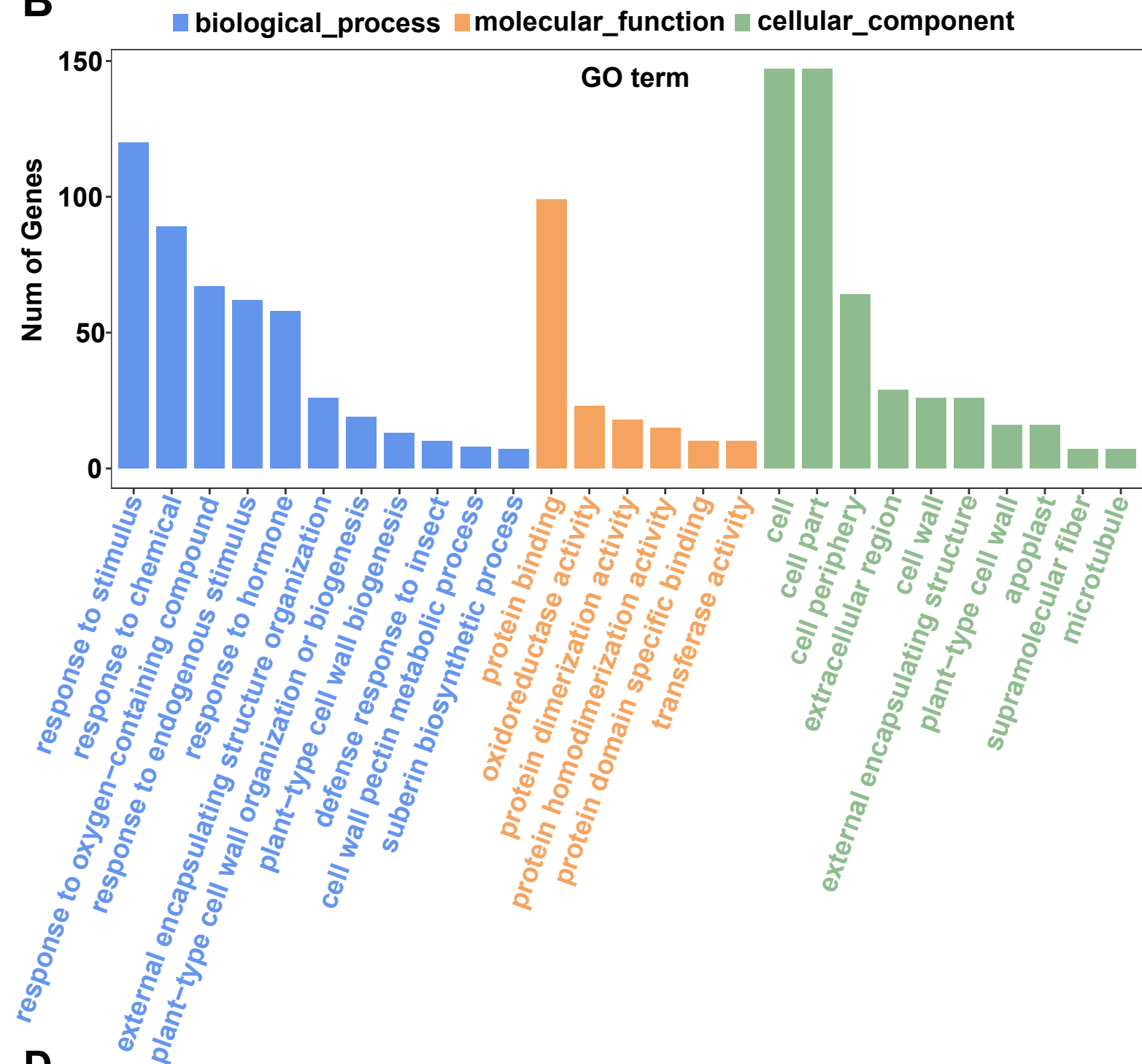

C

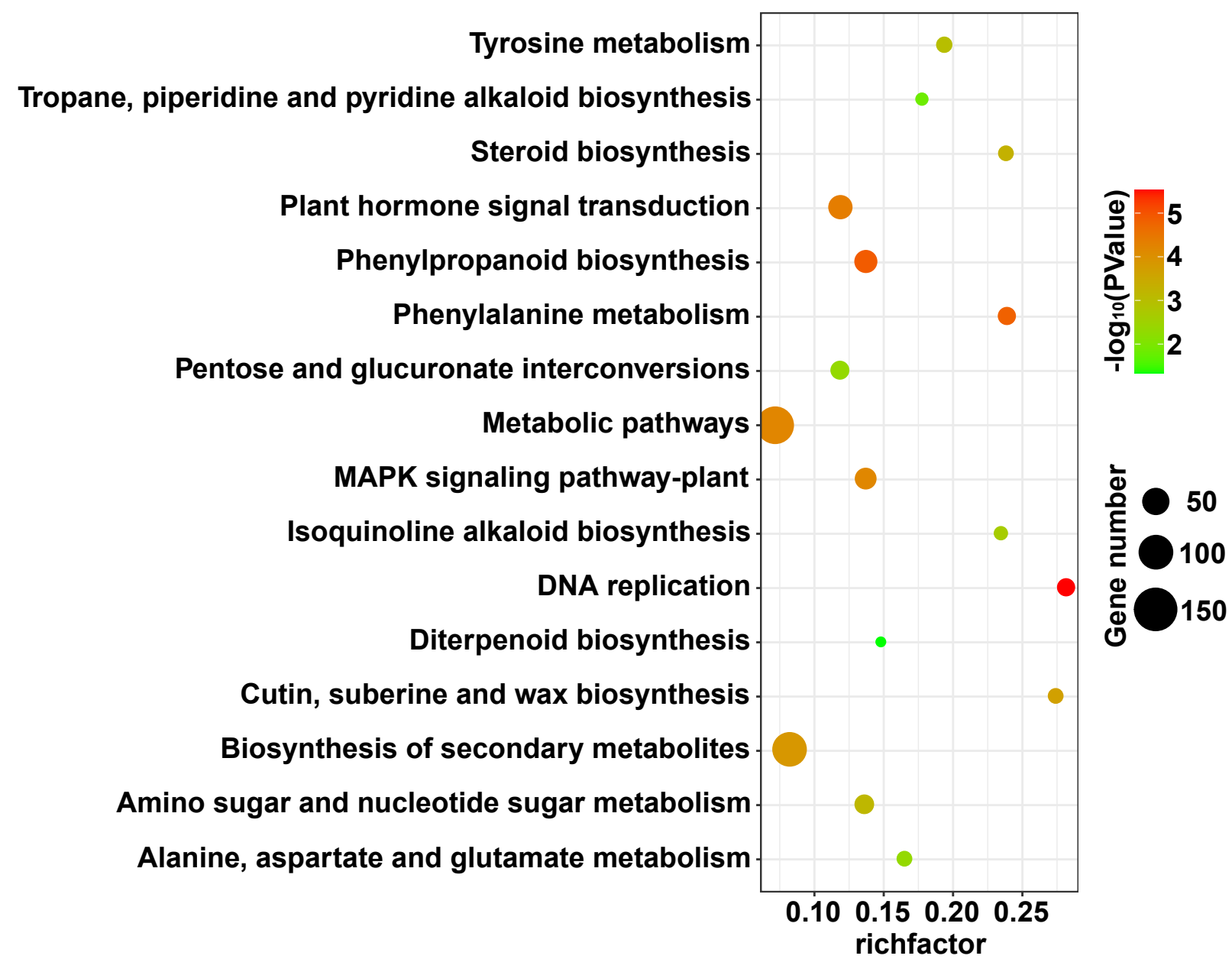

D

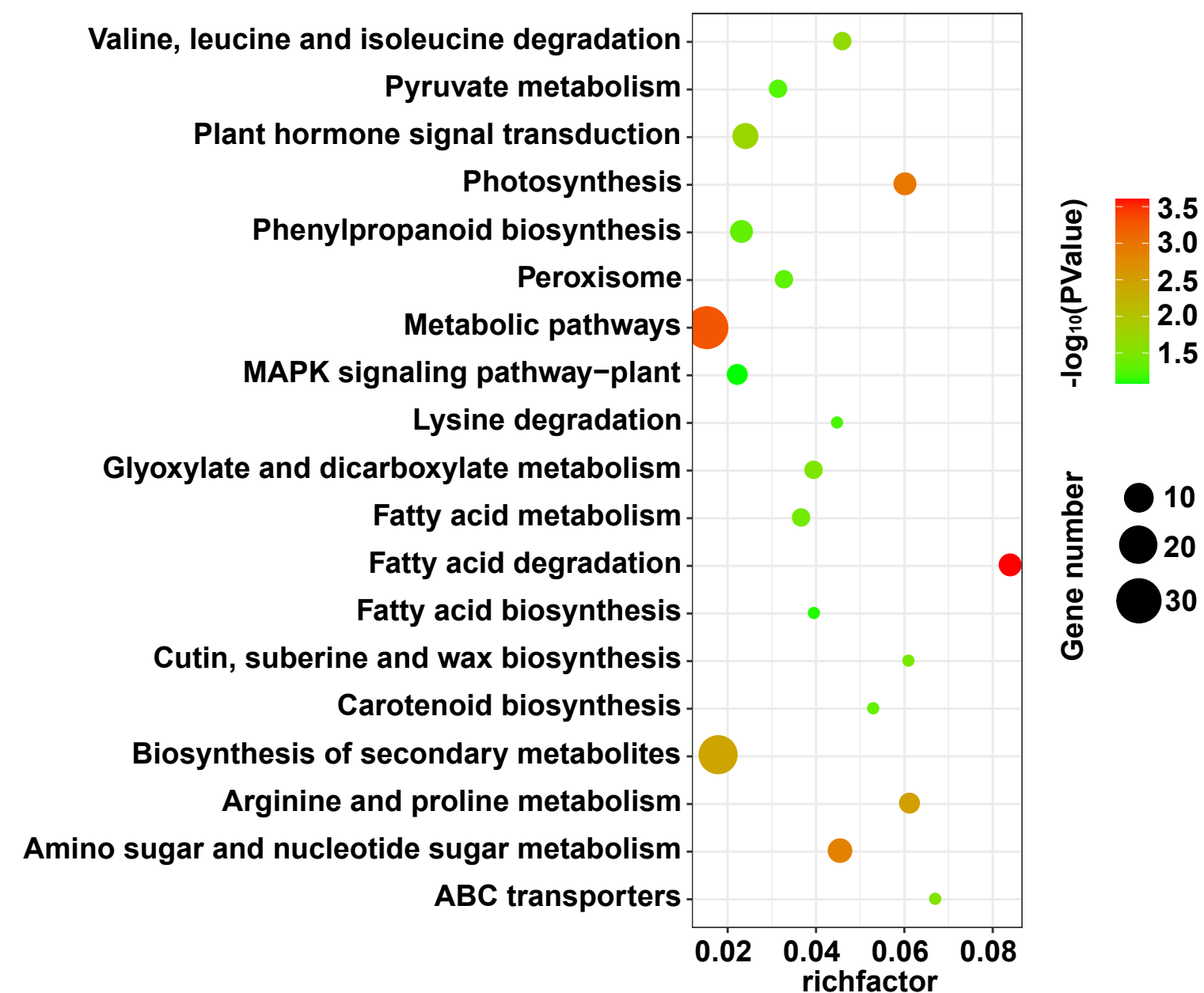

Supplement: Supplementary file 1 [file ijms-22-12238-s001.zip › Figure S2.pdf]
